# Nitrogen Fertilization Optimizes the Physicochemical Properties of Cactus Mucilage and the Biopolymeric Films Produced

**DOI:** 10.1021/acsomega.5c10740

**Published:** 2026-02-16

**Authors:** Lucas Vinícius Pierre de Andrada, Fred Augusto Louredo de Brito, Aline Lima Soares, Andréa Monteiro Santana Silva Brito, Thieres George Freire da Silva, Ivo Diego de Lima Silva, Gloria Maria Vinhas, Adriano do Nascimento Simões

**Affiliations:** † Federal Rural University of Pernambuco, Unidade Acadêmica de Serra Talhada, Serra Talhada, Pernambuco 56909-535, Brazil; ‡ Federal Rural University of Pernambuco, Recife, Pernambuco 52171-900, Brazil

## Abstract

In recent decades, increasing environmental concerns have driven interest in sustainable polymers, such as the mucilage derived from forage cactus (*Opuntia stricta*), which shows significant potential for food applications and the production of biopolymeric films. To expand its industrial use, improvements in synthesis methods, additive incorporation, and agronomic practices are necessary. However, studies on the use of *O. stricta* in films produced under nitrogen fertilization remain scarce, underscoring the need for further research to fully explore its potential in the bioplastics industry. Cladodes of *O. stricta* fertilized with 50, 150, 300, and 450 kg N ha^–1^ were washed, peeled, and ground with ethanol (99.8% P.A.) at a 2:3 ratio (parenchyma/alcohol), resulting in a dried mucilage powder used for analysis and film formulation. Lower nitrogen supply resulted in the highest cladode yield and produced mucilage with lower electrical conductivity, reduced sodium and potassium content, and a higher concentration of phenolic compounds, making it more suitable for antioxidant food applications. Films made from this mucilage demonstrated greater transparency, higher luminosity, increased stiffness, and reduced moisture content and water solubility. XRD and SEM analyses revealed a more crystalline and homogeneous structure, resulting in improved mechanical resistance. These outcomes indicate that nitrogen availability directly modulates mucilage composition and, consequently, the structural integrity and barrier properties of the films. The findings identify low nitrogen fertilization as a cost-effective and environmentally favorable strategy for producing consistent, high-quality biopolymeric films with potential applications in sustainable packaging.

## Graphical abstract

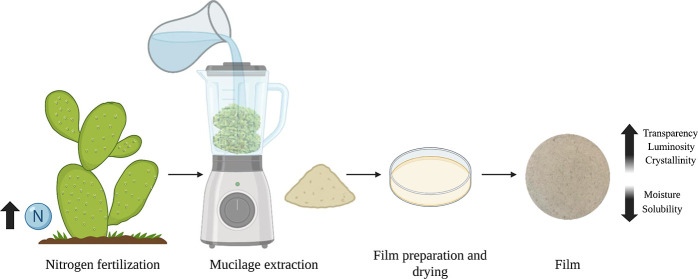

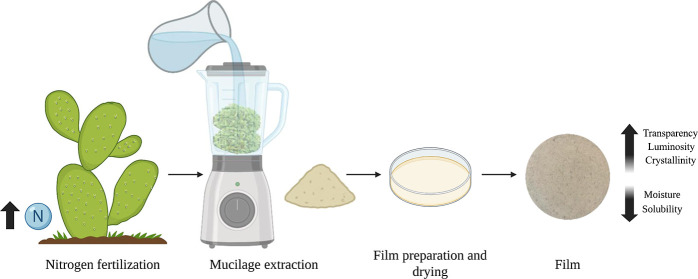

## 1 Introduction

In recent decades, there has been a growing awareness of the need to reduce the environmental impact of petroleum-derived plastics. This awareness has driven the demand for sustainable materials designed to decrease the consumption of nonrenewable resources during production. Consequently, the use of natural polymers has increased significantly, emerging as one of the most promising solutions to achieve sustainable development goals. These polymers offer a viable alternative to petrochemical-based plastics, promoting environmentally friendly materials across various industrial sectors.^1^

In this context, the valorization of agricultural residues and the extraction of biopolymers directly from biomass, such as polysaccharides, proteins, and lipids, have garnered considerable attention. This also includes biopolymers produced from yeast, algae, or bacterial fermentation biomass.^2^ These materials have attracted interest for a wide range of applications, including medical devices, food packaging, and biopolymeric films. Despite recent advancements in large-scale biobased polymer production, there remains ongoing interest in optimizing their industrial exploitation and improving the properties of the resulting biomaterials.^3^

Among the polysaccharides used in the synthesis of films and coatings is mucilage, a viscous substance obtained from various plant parts and species, such as cacti.^4^ The prickly pear cactus (*Opuntia stricta* (Haw.)), a member of the Cactaceae family, synthesizes mucilage in its tissues. It is widely used as animal feed in northeastern Brazil due to its high drought resistance, which results from its ability to store water in its tissues. This species has become an important raw material for formulating biodegradable films and coatings, although these still present disadvantages compared to conventional plastics.^5^ Consequently, considerable attention has been devoted to enhancing mucilage-based biopolymeric films to broaden their applications across various industries. This improvement can be achieved by modifying the synthesis process and incorporating different additives, such as micro and nanoparticles, plasticizers, and active agents, as well as by implementing preharvest treatments in the prickly pear cactus crop.^6^

It has been reported that prickly pear cactus crops extract high levels of nutrients from the soil during development, especially in soils typical of semiarid regions. This necessitates substantial inputs of nitrogen, potassium, sodium, calcium, and magnesium to achieve high productivity and biomass.^7^ According to Cunha and Gomes,^8^ the soils of the Brazilian semiarid region have low nitrogen availability for plants, making the use of external sources essential to increase prickly pear cactus biomass production.

The application of nitrogen in prickly pear cactus cultivation can enhance the synthesis of mucilage with improved properties, which supports the production of biopolymeric films and edible coatings, given nitrogen’s crucial role in plant metabolism. Nitrogen is an essential element for plants, involved in several vital processes such as growth, leaf area expansion, and biomass accumulation. Consequently, high nitrogen use efficiency (NUE) can lead to improved plant performance and favorable harvest outcomes.^9^ Various molecules, including amino acids, chlorophylls, and nucleic acids, contain nitrogen as a structural component and are essential for biological processes related to carbon and nitrogen metabolism, photosynthesis, and protein synthesis.^10^ Therefore, insufficient nitrogen availability can impede plant growth and development, as nitrogen also promotes root growth, enhancing the plant’s capacity to absorb nutrients.^11^

It has been reported that nitrogen application in prickly pear cactus cultivation enhances yield, chemical composition, and protein content, while also supporting the establishment and persistence of cultivated cladodes.^12^ Neto et al.^7^ observed that nitrogen fertilization increased plant height, width, and the number of cladodes, which may consequently promote greater mucilage synthesis in the plant’s parenchymal tissues. Increased mucilage production, accompanied by elevated protein content, can benefit the formulation of biopolymeric films. Nitrogen fertilization can influence specific mucilage properties, such as viscosity, elasticity, gel-forming ability, and protein content. These characteristics are critical in film production, as a matrix with higher viscosity and elasticity can yield stronger and more flexible films, while increased protein content may enhance their barrier properties.

Despite advances in characterizing cactus-derived mucilages, previous studies have primarily focused on *Opuntia ficus-indica* or have examined mucilage properties independently of agronomic practices. To date, no research has addressed how varying nitrogen fertilization levels influence the physicochemical composition of *O. stricta* mucilage and the resulting performance of its biopolymeric films. This distinction is important because *O. stricta* differs structurally and compositionally from other *Opuntia* species, and its response to nutrient availability may uniquely affect its film-forming capacity.

In this context, limited information is available regarding the potential uses of *O. stricta* for developing biopolymeric films under nitrogen fertilization. Although the literature highlights the potential of cactus mucilage in biofilm production, further characterization is necessary to improve the film-forming capabilities of the extracted polymers, thereby broadening their applicability in the bioplastics industry. Therefore, this study aimed to investigate the effect of nitrogen application on the properties of biopolymeric films formulated from *O. stricta* mucilage.

## 2 Material and Methods

### 2.1 Study Area

The plant material was obtained from the experimental site of the International Reference Center for Cactus and Other Forage Plants Studies (CentroRef) at the Federal Rural University of Pernambuco (UFRPE), Academic Unit of Serra Talhada (UAST), situated in Serra Talhada, PE, Brazil (7°59′S, 38°15′W; 431 m) (Figure 1A). The region’s climate is classified as BShw (hot semiarid, with dry winters and rainy summers) according to the Köppen climate classification.^13^ The area experiences an average air temperature of 26.6 °C, ranging from a minimum of 20.1 °C to a maximum of 32.9 °C, with an average annual precipitation of 642.1 mm and mean relative humidity of 62.5%.^14^ The meteorological variables were recorded using a data collection platform located approximately 10 m from the experimental site, operated by the National Institute of Meteorology. The corresponding data observed during the experimental period are shown in Figure 1B.

**Figure 1:**
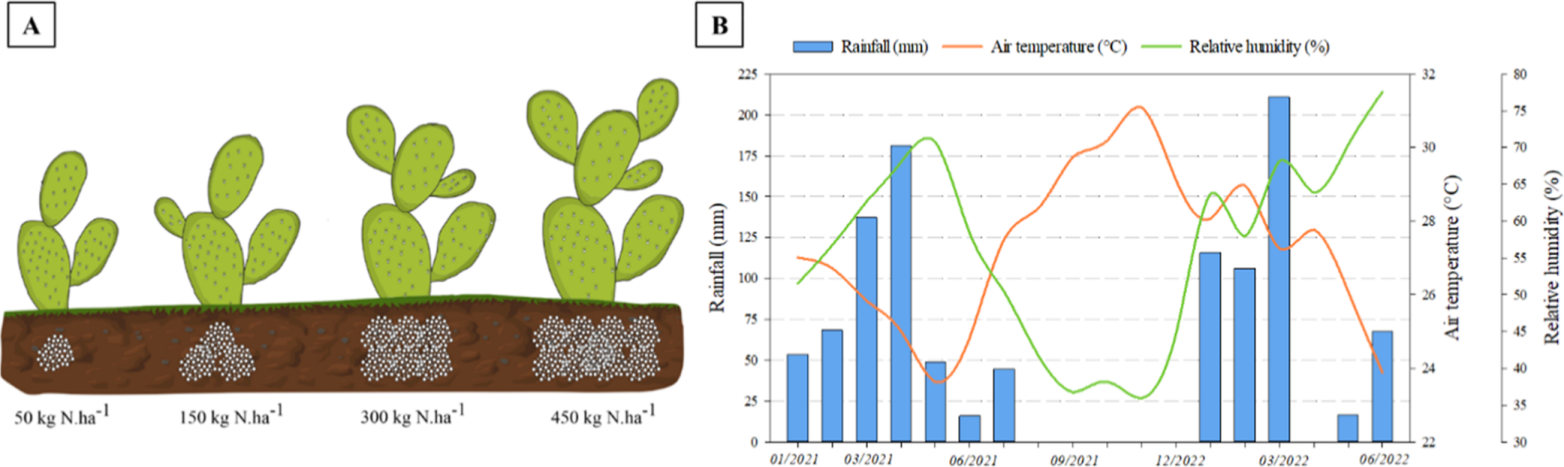
(A) Graphical representation of the experimental treatments: prickly pear cactus cladodes, *Opuntia stricta* (Haw.), subjected to 50, 150, 300, and 450 kg N ha^–1^ and (B) meteorological conditions during the experimental period.

### 2.2 Obtaining Cladodes and Mucilage Extraction

Prickly pear cactus cladodes of the “Mexican Elephant Ear” clone, *O. stricta* (Haw.), with an average size of 100 to 230 mm, were harvested from the middle third of the plant. These cladodes were subjected to four nitrogen fertilization doses: 50, 150, 300, and 450 kg N ha^–1^, as described by Alves et al.^15^ and Magalhães et al.^16^ (Figure 1). After harvesting, the cladodes were transported to the laboratory, where they were weighed, washed under running water, and peeled to remove the epidermis. The remaining aquiferous parenchyma was ground in a food processor (Philips Walita, RI7775, Barueri, Brazil) with ethyl alcohol (99.8% P.A.) at a 2:3 ratio (aquiferous parenchyma/alcohol) and homogenized, following the method of Panta de Araújo et al.^17^ Subsequently, successive washes with ethanol were performed to remove pigments and obtain a whitish precipitate. This precipitate was dried in an oven at 55 °C for 48 h. The resulting dried mucilage was then pulverized using a portable mill (Polespresso, Original Coffee flavor, Carapina da Serra, Brazil), producing a white powder that was used to determine the yield.

#### 2.2.1 Extraction Yield

Mucilage yield was calculated based on the fresh weight of the whole cladodes and the weight of the powder obtained after the extraction process, using the following formula

1 $$MY=\frac{Wf}{Wi}\times 100$$

where MY = mucilage yield in percentage (%), based on fresh weight; Wf = final weight of the powder obtained (g); and Wi = initial weight of all the cladodes (g).

### 2.3 Physicochemical Characterization and Technological Properties of the Mucilage

For the analyses of the mucilage (except for yield), the powder obtained was hydrated at a concentration of 4% w/v (4 g of powder per 100 mL of distilled water).

#### 2.3.1 Total Soluble Carbohydrates e Soluble Solids

The soluble carbohydrate content was determined according to the methodology described in Analytical Biochemistry.^18^ The mucilage was hydrated (2 mL) and centrifuged at 10,000 rpm and 4 °C for 21 min using a centrifuge (Hettich MIKRO 220, Berlin, Germany). Then, a 10 μL aliquot of the sample was mixed with 490 μL of deionized water, 500 μL of 5% phenol, and 2.5 mL of concentrated sulfuric acid (98.08%). After vortexing and resting for 10 min, absorbance readings were performed using a spectrophotometer (Libra S8, Biochrom, Cambridge, England) at 490 nm. Results were expressed as grams of soluble carbohydrates per 100 g of dry matter, quantified based on the equation obtained from the standard curve using glucose as a reference.

The total soluble solids content was measured using a benchtop refractometer (Instrutherm, RTD-95, São Paulo, Brazil). For the measurement, 1 mL of hydrated mucilage was used, and the results were expressed in °Brix.

#### 2.3.2 Total Titratable Acidity, pH, and Vitamin C

Total titratable acidity was determined according to the methodology described by Astello-García et al.^19^ Ten mL of previously hydrated mucilage were used. Two drops of 1% phenolphthalein were added to the solution, and the mixture was titrated with 0.1 N NaOH. Results were expressed as a percentage of citric acid, calculated using the following equation

2 $$TTA=\frac{n\cdot N\cdot Eq}{v}$$

where: TTA = total titratable acidity; *n* = volume of NaOH solution used in the titration (mL); *N* = molarity of the NaOH solution (0.1 N); eq = gram-equivalent of citric acid (64.02); *v* = sample volume (10 mL).

The hydrogen potential (pH) was measured using a pH meter (TECNAL, TEC-5, Piracicaba, Brazil) by directly immersing the electrode in the mucilage in a beaker.

Vitamin C content was determined using the Tillmans method, according to the methodology of the Adolfo Lutz Institute,^20^ which is based on the principle of titration. Results were expressed in milligrams of ascorbic acid per 100 g of dry matter (mg 100 g^–1^), calculated using the following formula

3 $$AA=\frac{V\cdot F\cdot 100}{A}$$

where: AA = ascorbic acid (mg 100 g); *V* = volume of Tillman’s solution used in the titration (mL); *A* = volume of the sample used (mL); *F* = Tillman’s solution factor.

The Tillman’s solution factor was calculated using the following formula

4 $$F=\frac{VitC}{ST}$$

where F = Tillman’s solution factor; VitC = amount of vitamin C solution used in the titration (mg); ST = volume of Tillman’s solution used (mL).

#### 2.3.3 Total Soluble Proteins and Total Phenolic Compounds

The total soluble protein content was determined using the Bradford method.^21^ Two mL of hydrated mucilage were centrifuged (Hettich, MIKRO 220, Berlin, Germany) at 10,000 rpm and 4 °C for 21 min. Then, 100 μL of the supernatant was mixed with 1000 μL of Bradford reagent. The tubes were vortexed (TECNAL, AP56, Araraquara, Brazil) and incubated at room temperature for 15 min. Absorbance was measured using a spectrophotometer (Biochrom, Libra S8, Cambridge, England) at 595 nm. Bovine serum albumin (BSA) was used as the external standard. Total soluble protein content was expressed as mg of soluble protein per 100 g of dry matter.

Total phenolic compound content was determined according to the methodology of Chandra & De Mejia.^22^ Two mL of hydrated mucilage were centrifuged (Hettich, MIKRO 220, Berlin, Germany) at 10,000 rpm and 4 °C for 21 min. Then, a 150 μL aliquot of the supernatant was combined with 100 μL of deionized water and 250 μL of Folin-Ciocalteu reagent (1 N). The mixture was vortexed (TECNAL, AP56, Araraquara, Brazil) and allowed to rest for 2 min. Subsequently, 500 μL of 20% (w/v) sodium carbonate was added, and the mixture was left to stand for an additional 10 min. Absorbance readings were then performed using a spectrophotometer (Biochrom, Libra S8, Cambridge, England) at 757 nm.

#### 2.3.4 Electric Conductivity and Sodium (Na^+^) and Potassium (K^+^) Content

Electrical conductivity was measured using a DDS-12DW microprocessor conductivity meter, with the sensor immersed directly in a beaker containing the mucilage samples. Results were expressed in mS cm^–1^. For sodium and potassium content analysis, test tubes containing 200 μL of mucilage were diluted with 9800 μL of water at a 1:50 ratio (mucilage/water), resulting in a final volume of 10 mL. The samples were filtered, and measurements were conducted using a flame photometer (B-462 MICRONAL). Results are expressed as μmol of Na^+^ or K^+^ per mL of mucilage.

#### 2.3.5 Water and Oil Holding Capacity

Water holding capacity (WHC) was determined using the method described by Alba and Maryna.^23^ Mucilage samples (0.2 g) were added to 10 mL of distilled water in Falcon tubes, left at room temperature for 1 h, and shaken for 5 s every 15 min. The samples were then centrifuged at 5000 rpm for 20 min. The supernatant was discarded, and the remaining material in the tubes was dried in an oven at 55 °C for 30 min to remove residual water.

Oil holding capacity (OHC) was measured following the methodology of Gan et al.^24^ Mucilage samples (0.1 g) were added to 10 mL of soybean oil in Falcon tubes and shaken at 200 rpm in an incubator (TECNAL, model TE-420) for 1 h. The mixture was then centrifuged at 5000 rpm for 15 min. The supernatant was discarded, and the precipitate was dried in an oven at 55 °C for 24 h. WHC and OHC were calculated, and the results were expressed as grams of water and oil adsorbed per gram of mucilage, respectively, as follows

5 $$WHC\;\left(gg^{-1}\right)=OHC\;\left(gg^{-1}\right)=\frac{weight\;of\;the\;mucilage\;after\;drying}{Initial\;weight\;of\;mucilage}$$

### 2.4 Formulation of Biopolymeric Films

For film preparation, the methodology described by was followed. The powder obtained from the extraction was hydrated at a concentration of 4% (w/v) to form an emulsion. Glycerol was added to this emulsion as a plasticizer at a concentration of 40% (v/w) relative to the powder, under constant heating at 70 °C. The resulting mixture was then poured into Petri dishes and dried in an oven at 55 °C for 24 h to form the films.

### 2.5 Physicochemical, Optical, Structural, Mechanical, and Thermal Characterization of the Films

#### 2.5.1 Fourier-Transform Infrared Spectroscopy (FTIR) and X-ray Diffraction (XRD)

Spectral analyses in the mid-infrared region were conducted using a Fourier-transform infrared (FTIR) spectrophotometer (Frontier, PerkinElmer) equipped with an attenuated total reflectance universal accessory (UATR). Spectra were recorded over the range of 4000–400 cm^–1^, with a resolution of 8 cm^–1^ and 8 scans per sample. Air was used as the background reference, and measurements were taken directly on the film placed on the diamond crystal. X-ray diffraction (XRD) analyses were performed using an X-ray diffractometer (Rigaku Miniflex 600, Japan) operating at 40 kV and 15 mA. Spectra were collected at room temperature over a 2θ range of 2° to 50°, with a scanning rate of 2°/min. The films were analyzed under these conditions.

#### 2.5.2 Thickness, Transparency and Color

Film thickness (mm) was measured at 10 random points using a digital micrometer with a resolution of 1 μm, and the average value was calculated.^25^ Transparency was assessed using rectangular film segments placed in cuvettes of a spectrophotometer (Biochrom, Libra S8, Cambridge, England), positioned perpendicular to the light path to measure absorbance at 600 nm. An empty cuvette served as the blank. Transmittance (%) at 600 nm was calculated as Tr600 = 10^–Abs^ × 100, and transparency was expressed as a percentage, calculated according to the following formula

6 $$T=\frac{Tr600}{x}$$

where: *T* = transparency; Tr_600_ = transmittance at 600 nm; *x* = thickness of the film (mm).

Color measurements were conducted using a portable colorimeter (RS-232 with serial output, model RGB-1002). The device provided RGB color parameters, which were then converted to CIE Lab* values using the online tool at http://www.easyrgb.com/index.php?X=CALC#Result, with D65 as the standard illuminant (daylight) and a 2° observer angle. The luminosity (L*) parameter was obtained directly from the colorimeter and required no conversion. The visual color of the samples was interpreted based on the L*, a*, and b* values.

#### 2.5.3 Moisture Content, Water Solubility and Water Vapor Permeability

Moisture content was determined using films measuring 2.0 × 2.0 cm, which were first weighed and then dried in an oven at 55 °C for 24 h. The samples were subsequently weighed daily until a constant mass, corresponding to the dry sample mass, was reached. Solubility was assessed using 2.0 × 2.0 cm films. These were dried in an oven at 55 °C for 24 h, cooled to room temperature in a desiccator, weighed, and then immersed in 50 mL of distilled water at 25 °C for 30 min. After this period, the undissolved fragments were dried again in the oven at 55 °C for 24 h, cooled in a desiccator, and weighed. The final weights of the fragments were used to calculate the moisture content and water solubility of the films, expressed as percentages and determined using the following formula

7 $$MC=WS=\frac{Wi-Wf}{Wi}\times 100$$

where MC = moisture content; WS = water solubility; Wi = initial weight of the films (g); and Wf = final weight of the films.

Water vapor permeability (WVP) was determined according to the ASTM method. Film samples were placed over beakers containing 70 g of calcium carbonate (CaCO_3_), maintaining an approximate 10 mm gap between the carbonate and the film. The beakers were then stored in a desiccator, with temperature and relative humidity controlled at 25 °C and 75% RH, respectively. WVP was calculated based on the weight gain of the beakers, with the slope of weight change over time determined by linear regression (*R* ^2^ > 0.99). WVP (g mm^–1^ m^–2^ d^–1^ kPa) was calculated using the following formula

8 $$WVP=\frac{WVTR\cdot X}{\Delta p}$$

where WVTR = water vapor transmission rate (g m^–2^ d^–1^) defined as the slope (g d^–1^) divided by the transfer area (m^2^); *x* = film thickness (mm); Δ*p* = difference in water vapor partial pressure across the film; and Δ*p* = *p* (RH2 – RH1) = 2.38 kPa, where *p* is the water vapor saturation pressure at 25 °C, RH2 = 75% and RH1 = 0%.

#### 2.5.4 Scanning Electron Microscopy (SEM)

The microstructural morphology of the film surfaces was analyzed using scanning electron microscopy (SEM). The samples were mounted on stubs and coated with gold using a sputter coater (DENTON VACUUM, DESK V model). They were then examined with a TESCAN SEM (VEGA3) equipped with a tungsten filament, and images were captured at an acceleration voltage of 20.0 kV.

#### 2.5.5 Mechanical Properties: Tensile Strength, Elongation at Break and Young’s Modulus

The mechanical properties of the films were evaluated using a tensile test performed on a TA.XT Plus texturometer (TA Instruments, New Castle, USA), following the methodology described in ASTM D882-12 (2012). Prior to testing, film samples, cut to 10 cm × 2.5 cm, were conditioned in a saturated NaBr saline solution (58% relative humidity) for 5 days at 25 ± 2 °C. Testing conditions were set as follows: an initial probe distance of 8 cm and a constant crosshead speed of 1.0 mm/s, applied until film rupture. Tensile strength (MPa) was calculated by dividing the maximum force at break by the initial cross-sectional area of the film. Elongation at break was expressed as the maximum extension of the film relative to its original length (%). Young’s modulus (MPa) was determined as the ratio of the longitudinal stress applied to the film to the resulting elastic strain.

#### 2.5.6 Water Angle Contact

The contact angle of a water droplet on the film surface was measured using an optical tensiometer (Optical Tensiometer, Finland). The films were placed on a dedicated holder, and a droplet of Milli-Q water was gently deposited onto the sample surface using a precision syringe. Images were captured every second over a 10 s interval. The angle formed between the film surface and the tangent to the water droplet was determined using the equipment’s integrated software.^26^

### 2.6 Statistical Analysis

The field experiment was conducted using a randomized complete block design (RCBD) with five replications. Data were tested for normality and analyzed using regression analysis with R x64 3.4.0 software. Graphs were generated using SigmaPlot version 14 (Systat Software Inc., 2020) and OriginPro.

## 3 Results and Discussion

### 3.1 Yield and Physicochemical Properties of Prickly Pear Cactus Mucilage

The agroindustrial yield, total soluble solids, vitamin C, total soluble proteins, and total soluble carbohydrates decreased with increasing nitrogen fertilization (*p* < 0.05) (Table 1; Figure 2). Conversely, increasing nitrogen application in the soil led to higher citric acid content, pH, electrical conductivity, as well as elevated levels of Na^+^, K^+^, water and oil retention capacity, and total phenolic compounds (Table 1; Figure 2).

**Table 1:** Yield (%), Total Soluble Solids (°Brix), Total Titratable Acidity (% Citric Acid), Vitamin C (mg 100 g^–1^ DM), pH, Electrical Conductivity (mS cm^–1^), Sodium (Na^+^) and Potassium (K^+^) Content (mg 100 g^–1^ DM), Total Soluble Proteins (mg 100 g^–1^ DM), Total Soluble Carbohydrates (mg 100 g^–1^ DM), Total Phenolic Compounds (mg Gallic Acid 100 g^–1^ DM), and Water (g Water per g Mucilage) and Oil (g Oil per g Mucilage) Holding Capacity in the Mucilage of *Opuntia stricta* (Haw.), Subjected to Nitrogen Fertilizations of 50, 150, 300, and 450 kg N ha^–1^

	nitrogen level (kg N ha^–1^)				
quantification	50	150	300	450	equation
cladode yield	3.18	2.73	2.38	2.55	CY = 0.1534*x* ^2^ – 0.9909*x* + 4.0427
parenchyma yield	42.91	44.43	42.72	37.75	PY = −1.6206*x* ^2^ + 6.3819*x* + 38.157
total soluble solids	1.43	1.06	1.16	0.76	SS = −0.0083*x* ^2^ – 0.1483*x* + 1.5417
total titratable acidity	1.34	2.01	2.68	1.67	TTA = −0.4204*x* ^2^ + 2.2679*x* – 0.5908
vitamin C	2.66	2.50	2.83	2.33	VC = −0.0833*x* ^2^ + 0.35*x* + 2.3333
pH	5.25	5.24	5.31	5.74	pH = 0.1108*x* ^2^ – 0.4018*x* + 5.5608
electric conductivity	460.56	1233.33	1359.66	1690.66	EC = −110.44*x* ^2^ + 933.87*x* – 320.31
sodium content (Na^+^)	65.23	65.23	74.79	65.23	SC = −2.3876*x* ^2^ + 12.893*x* + 53.302
potassium content (K^+^)	568.38	937.87	937.87	1139.41	PC = −41.987*x* ^2^ + 381.24*x* + 257.68
water holding capacity	15.50	13.88	12.28	19.88	WHC = 2.3039*x* ^2^ – 10.365*x* + 24.021
oil holding capacity	12.16	9.55	9.74	15.15	OHC = 2.0025*x* ^2^ – 9.0984*x* + 19.384

**Figure 2:**
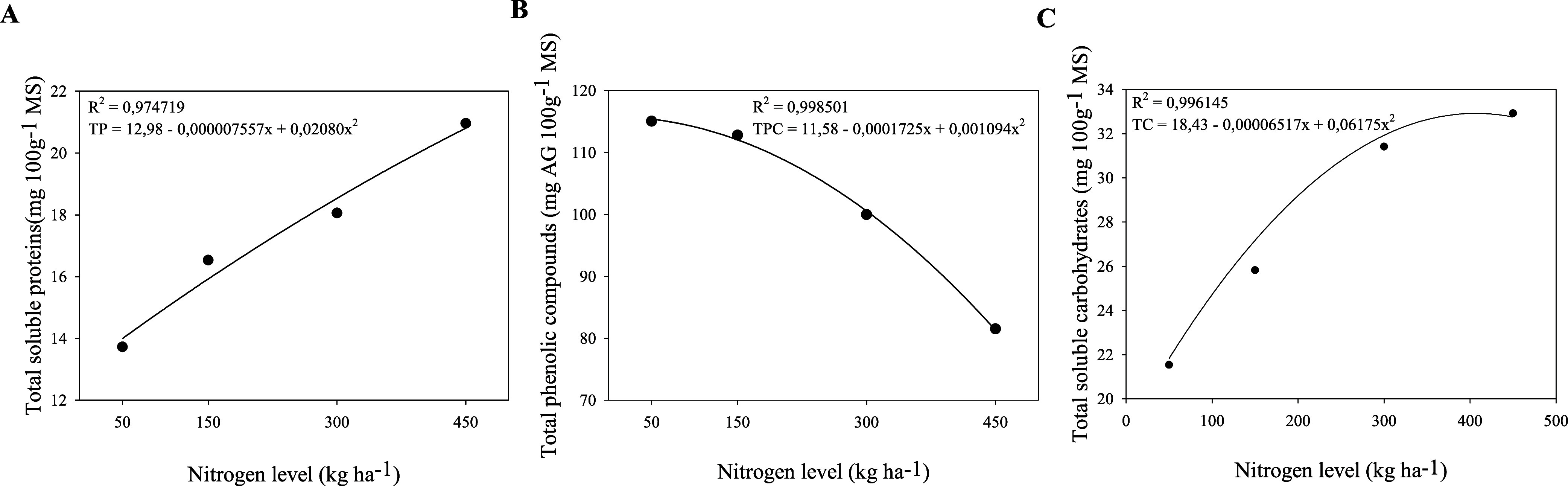
Total soluble proteins (mg 100g^–1^ MS) (A), total phenolic compounds (mg AG 100g^–1^ MS) (B) and total soluble carbohydrates (mg 100 g^–1^ MS) (C) in the mucilage of *Opuntia stricta* (Haw.), subjected to nitrogen fertilizations of 50, 150, 300, and 450 kg N ha^–1^.

The yield of mucilage extraction is a key factor limiting its industrial-scale application. In the present study, a trend of decreased yield with increased nitrogen fertilization was observed (Table 1), suggesting that higher nitrogen levels negatively affect mucilage yield. The yield obtained at the lowest nitrogen dose was 3.18% (Table 1), substantially higher than those reported by Espino-Díaz et al.^27^ and Pinheiro et al.,^5^ who reported yields of 0.68% and 0.41% for ethanolic extraction of *O. ficus-indica* and *O. stricta* mucilages, respectively. Since mucilage is synthesized as an adaptive response to water stress, especially in arid and semiarid environments, increased nitrogen fertilization may have enhanced root system development and improved water and nutrient absorption efficiency.^28^ This likely reduced the physiological demand for mucilage production as a water storage medium in cactus tissues, resulting in decreased synthesis and extraction. An additional explanation for the decline in mucilage yield at higher nitrogen levels is the potential occurrence of physiological stress or nitrogen saturation. Although nitrogen is essential for photosynthesis and biomass accumulation, excessive amounts can disrupt the carbon–nitrogen balance and shift metabolic priorities, potentially reducing the allocation of photoassimilates to polysaccharide synthesis.^29^ Achieving higher mucilage yields with reduced nitrogen application can lower agricultural input costs, thereby reducing overall production costs in forage cactus cultivation, particularly for biopolymeric films production.

On the other hand, the levels of Na^+^ and K^+^ in the mucilage increased with higher nitrogen concentrations in the soil (Table 1), confirming greater absorption of these ions at 450 kg N ha^–1^. Nitrogen may also enhance the efficiency of the root system in absorbing various ions, promoting significant accumulation of these elements in the plant’s mucilage. These results are important because the remarkable mineral composition of cactus mucilage makes it ideal as a nutritional additive in food formulations.^30^ Increased nutrient absorption may also alter the ionic balance of the mucilage, affecting pH and electrical conductivity, which were also higher in mucilage from cladodes fertilized with the highest nitrogen dose (Table 1). These elevated conductivity and pH values at the highest fertilization level are unsuitable for film formulation based on mucilage, as soluble ions can interfere with the formation of stable polymer networks essential for the structural integrity of the films. They may also cause compatibility issues with other components used in the film formulations, leading to phase separation, reduced mechanical strength, and decreased film quality.^31^

Nitrogenous compounds, such as proteins and amino acids, exhibit a strong affinity for both water and oil, which may explain the increased water (19.88 g g^–1^) and oil (15.15 g g^–1^) retention observed in mucilage from plants treated with 450 kg N ha^–1^ (Table 1). Several studies have reported that mucilages have a greater capacity to bind hydrophilic substances compared to hydrophobic ones. For example, the mucilage of *Opuntia dillenii* demonstrated a higher water holding capacity (4.0 g g^–1^) than oil holding capacity (2.0 g g^–1^).^32^ Similarly, Bayar et al.^33^ water and oil retention values of 7.81 g g^–1^ and 1.34 g g^–1^, respectively, in *O. ficus-indica* mucilage. In the present study, the increased biomass and chemical composition changes induced by nitrogen likely enhanced the viscosity and molecular interaction capacity of the mucilage, resulting in greater water retention. The polysaccharides in the mucilage possess hydrophilic properties, which accounts for the lower oil retention.^34^ However, the oil holding capacity observed was higher than values reported in other studies, potentially improving the compatibility of biopolymeric films formulated with oily substances. This facilitates the efficient incorporation of lipophilic compounds, such as waxes, thereby enhancing the hydrophobic properties of the resulting films. Furthermore, the high oil retention suggests that the mucilage could improve the texture of food products.^35^

Increased nitrogen fertilization raised the protein content in the mucilage (Figure 2A). This finding indicates that the applied nitrogen contributed to the assimilation of nitrogen in organic form, which is essential for plant growth and development. Conversely, higher nitrogen fertilization reduced the levels of phenolic compounds in the mucilage (Figure 2B). Phenolic compounds are important secondary metabolites produced by plants in response to environmental stresses such as herbivory and water deficit. Their roles as antioxidants and antimicrobial agents provide the plant with defense mechanisms, enhancing its survival and resistance under adverse conditions. Adequate nitrogen supply through fertilization may have alleviated stress in the cactus, resulting in a reduction in total phenolic compound production in the mucilage.^36^ In this context, the plant allocated fewer resources to the synthesis of these compounds, as protection against environmental stresses was improved by the enhanced growth conditions provided by nitrogen fertilization.

An increase in carbohydrate content was observed as a result of nitrogen fertilization (Figure 2C). Nitrogen is essential for the biosynthesis of amino acids and proteins, which are crucial for plant growth and development. It also enhances photosynthetic activity by promoting the synthesis of chlorophyll and photosynthetic enzymes. Improved photosynthesis leads to increased carbon fixation, resulting in higher production of sugars and carbohydrates that are stored in the plant’s mucilage.^37^ Furthermore, the integration of nitrogen and carbon metabolism enables more efficient energy use, redirecting resources toward carbohydrate synthesis. This results in greater accumulation of polysaccharides in the mucilage, which aids in water retention and adaptation to arid environments.^38^ Sources with high carbohydrate content are advantageous because, as food additives, these substances are versatile and widely used as emulsion stabilizers, texturizers, and fat replacers.^39^ In biopolymeric packaging systems, the hydrophilic nature of carbohydrates can enhance the films’ ability to absorb and retain moisture, thereby improving their flexibility and elasticity. However, excessive moisture retention may compromise the dimensional stability and tensile strength of the films, making them more susceptible to degradation under humid conditions.^40^

Previous research on *Opuntia* polysaccharides suggests that nutrient supply may alter carbohydrate metabolism, affecting the relative abundance of arabinose, galactose, and xylose-rich fractions, or modifying polymer chain length. Such changes can influence branching patterns, the degree of polymerization, and intermolecular interactions, ultimately impacting viscosity, gel formation, and network organization within the mucilage.^41^ These structural modifications are also known to affect water affinity, ionic interactions, and compatibility with plasticizers in film matrices. Therefore, future investigations should include detailed compositional and molecular characterization to elucidate the biochemical mechanisms by which nitrogen fertilization influences mucilage structure and its film-forming capacity.

### 3.2 Physicochemical, Structural, Thermal, and Optical Properties of the Films

#### 3.2.1 Fourier-Transform Infrared Spectroscopy (FTIR) and X-ray Diffraction (XRD)

Infrared spectra of biopolymeric films are crucial for characterizing the chemical bonds and functional groups present in the biomaterial. The peaks observed were more pronounced in films derived from cacti fertilized with the highest nitrogen dose, showing a trend of reduced peak intensity at lower doses (Figure 3A). The absorption peak around 3331 cm^–1^ is associated with vibrations of −OH groups from alcohols and carboxylic acids involved in intermolecular hydrogen bonding, indicating the films’ affinity for water molecules.^42^ This contributes to the higher moisture content and solubility observed in films from 450 kg N ha^–1^ (Figure 4A and B). The asymmetric C–H stretching vibrations corresponding to the peak at 2921 cm^–1^ indicate the presence of cellulose in the film formulation,^43^ which may explain the higher tensile strength and elongation at break observed at 450 kg N ha^–1^ (Figure 6A and B). Two bands near 1700 and 1640 cm^–1^ are attributed to C=O vibrations of carboxyl groups and COO^–^ stretching characteristic of carboxylic acid salts present in the mucilage, respectively.^44^ Additionally, a set of peaks between 1430 and 1240 cm^–1^ corresponds to C–H or O–H vibrations.^42^ The most intense peak, observed around 1040 cm^–1^, indicates the presence of polysaccharides, represented by C–O–C or C–O–H vibrations,^45^ consistent with the higher carbohydrate content observed in films from cacti subjected to the highest nitrogen dose (Figure 2C).

**Figure 3:**
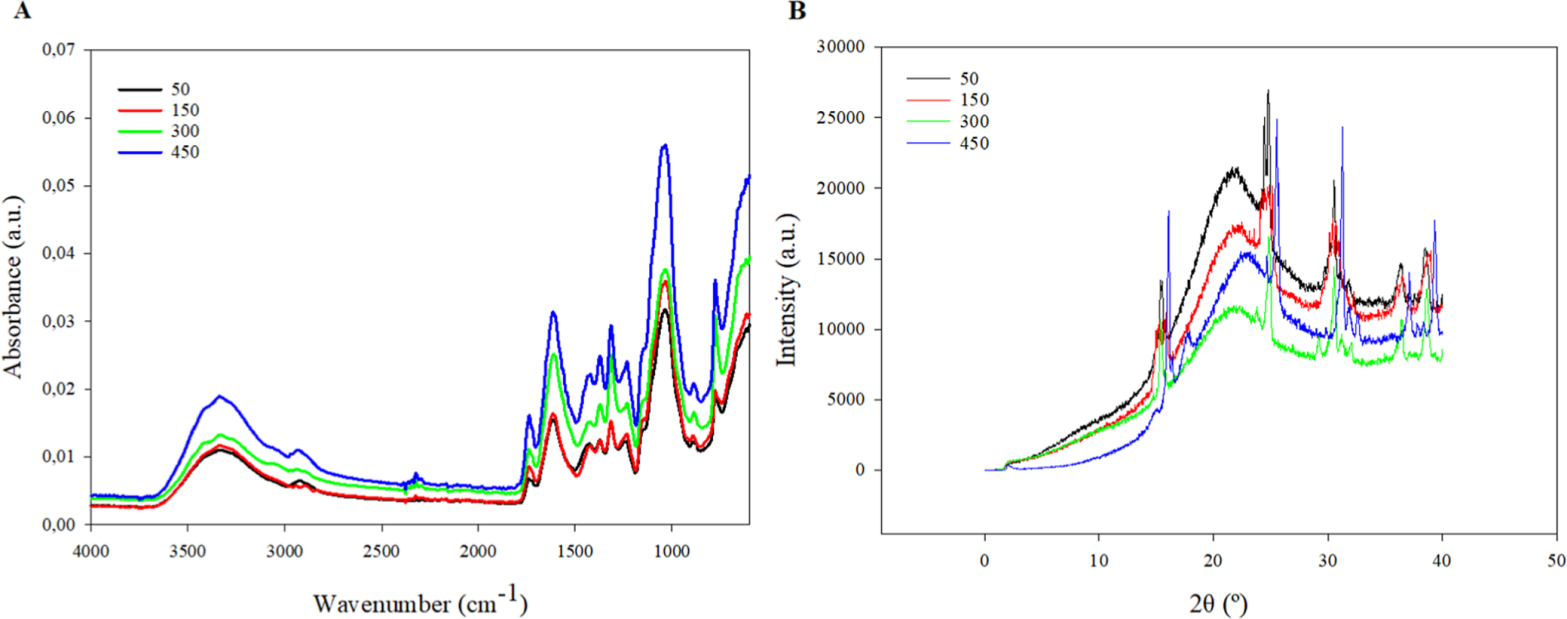
FTIR spectrum (A) and X- ray diffractometry (B) of films based on the mucilage of *Opuntia stricta* (Haw.), subjected to nitrogen fertilization at 50, 150, 300 e 450 kg N ha^–1^.

**Figure 4:**
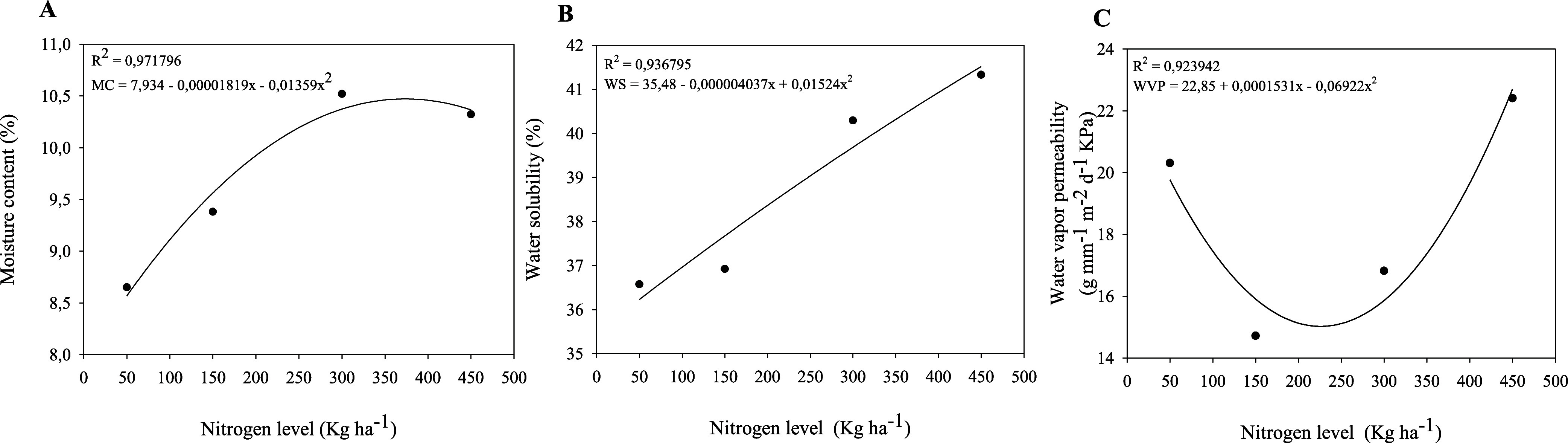
Moisture content (%) (A), water solubility (%) (B) and water vapor permeability (g mm^–1^ m^–2^ d^–1^ kPa) (C) of films based on the mucilage of *Opuntia stricta* (Haw.), subjected to nitrogen fertilization at 50, 150, 300 e 450 kg N ha^–1^.

Crystallinity is a critical factor for polymers in various applications, including microencapsulation and the food packaging industry. Materials with a higher degree of crystallinity are preferred because they are less hygroscopic and demonstrate greater stability during storage.^34^ In contrast, amorphous substances tend to absorb water, which can compromise food structure, promote microbial growth, and cause nutrient degradation. Films treated with 50 kg N ha^–1^ exhibited higher-intensity peaks in the X-ray diffraction spectra (Figure 3B), indicating greater crystallinity and mechanical strength. The lower nitrogen concentration resulted in a more ordered and crystalline structure, with sharp peaks observed at 2θ = 15°, 25°, and 30°. As the nitrogen doses increased, there was a progressive decrease in peak intensity, suggesting reduced crystallinity of the materials. The increased synthesis of nitrogenous compounds in the mucilage at higher nitrogen doses may lead to a more amorphous and disordered structure in the films, thereby decreasing the intensity of the peaks in the XRD spectra.

#### 3.2.2 Thickness, Transparency and Color

Increasing nitrogen doses tended to produce thicker films, with thickness increasing up to 300 kg N ha^–1^. The thinnest films, observed at 50 and 450 kg N ha^–1^, exhibited higher transparency, reflecting the inverse relationship between thickness and transparency. Lightness was highest at 50 kg N ha^–1^, while films formed at 150 kg N ha^–1^ were the darkest observed. Films from 50 kg N ha^–1^ demonstrated a more compact structure, greater flexibility, and good transparency (>10% mm^–1^) (Table 2). These films were more transparent than those reported for *Opuntia* films by por González Sandoval et al.^46^ and Pinheiro et al.,^5^ who documented transparencies of 7.43% mm^–1^ and 4.73% mm^–1^, respectively. These findings suggest that using low levels of fertilizer does not compromise film quality, offering significant savings in agricultural inputs. Moreover, higher transparency is a desirable property in packaging design, as it enhances product visibility, adds aesthetic value, and facilitates consumer acceptance, particularly in applications requiring visual appeal and the perception of freshness.

**Table 2:**
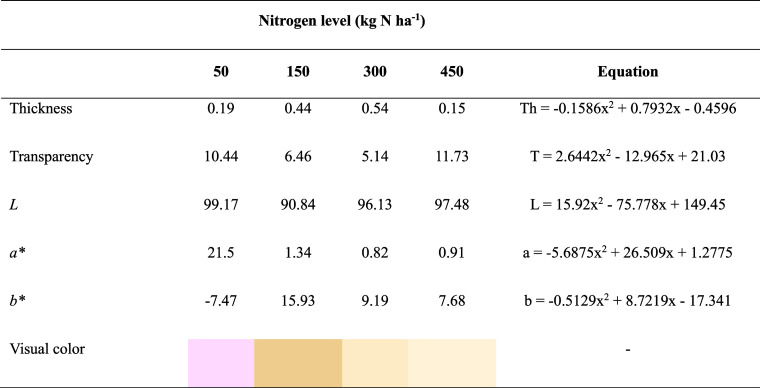
Thickness (mm), Transparency (% mm^–1^) and Color of Films Based on the Mucilage of *Opuntia stricta* (Haw.), Subjected to Nitrogen Fertilization at 50, 150, 300 e 450 kg N ha^–1^

a Color parameters indicate that nitrogen fertilization levels significantly influenced the coloration of the films. The parameter a*, which measures the variation from green (negative values) to red (positive values), was highest in films obtained with 50 kg N ha^–1^ (Table 2), indicating a slight pink hue, while the other treatments showed values close to 1, suggesting a more neutral tone. The parameter b*, representing the transition from blue (negative values) to yellow (positive values), increased markedly with higher nitrogen fertilization, rising from 7.47 to 15.93, indicating the development of yellowish tones in films with elevated nitrogen levels. These color variations may be associated with changes in mucilage composition, particularly in the proportion of phenolic compounds and pigments, which affect the final appearance of the films. From an application perspective, lighter films with less yellowing, such as those produced under lower fertilization, are generally more desirable for food packaging, as they enhance product visibility and aesthetic appeal.

#### 3.2.3 Moisture Content, Water Solubility and Water Vapor Permeability

Films derived from plants subjected to higher nitrogen fertilization exhibited increased moisture content, water solubility, and water vapor permeability (Figure 4). Certain applications of biopolymeric films, such as food packaging, require low solubility and moisture content to enhance the integrity and water resistance of food products. These parameters are influenced by the hydrophobic and hydrophilic components of the film. It was observed that films from plants fertilized with higher nitrogen doses, particularly at 300 kg N ha^–1^, showed the highest moisture content (10.52%) (Figure 4A), possibly due to greater water retention resulting from a more porous film structure. In contrast, films from plants fertilized with 50 kg N ha^–1^ exhibited lower moisture content (8.65%), reflecting a more compact structure with reduced water permeability. The moisture content of these films was lower than that reported by Gheribi et al.^47^ and Brito et al.,^48^ who formulated *Opuntia* films with 14% and 9.53% moisture, respectively. Water solubility of the films was also influenced by the fertilization dose. Films from plants fertilized with 50 kg N ha^–1^ were less soluble in water (36.57%) (Figure 4B), which is advantageous for applications requiring stability in humid environments. These films were also less soluble than films based on *O. ficus-indica* and *O. stricta* grown without nitrogen fertilization, as reported by Sandoval et al.^46^ and Brito et al.,^48^ who observed solubility values of 91.05% and 96.04%, respectively. Therefore, the data suggest that films derived from cacti subjected to the lowest nitrogen fertilization dose are suitable for applications in the packaging industry.

Water vapor permeability is defined as the amount of water vapor transmitted through a film under specific temperature and relative humidity conditions. It is a critical property for biopolymeric films used in food packaging, as effective barrier properties can prevent food oxidation and microbial spoilage, thereby extending shelf life.^42^ Pinheiro et al.^5^ produced films from *O. stricta* and reported a permeability of 31.93 g mm^–1^ m^–2^ d^–1^ kPa. In the present study, films produced with 150 and 300 kg N ha^–1^ exhibited lower permeability rates (14.72 and 16.82 g mm^–1^ m^–2^ d^–1^ kPa, respectively) (Figure 4C), indicating a more effective moisture barrier. This reduction in permeability was attributed to the greater thickness of these films (Table 2), which decreases the rate of moisture transmission through the material’s surface. This characteristic is essential for food preservation, as it minimizes moisture exchange between the product and the environment, thereby prolonging shelf life. However, despite these benefits, the films were brittle and poorly formed, rendering them unsuitable for industrial applications despite their lower permeability. In this context, films produced with 50 kg N ha^–1^, which exhibited a permeability of 20.31 g mm^–1^ m^–2^ d^–1^, are more appropriate for maintaining food quality in packaging applications.

#### 3.2.4 Visual Appearance and Scanning Electron Microscopy (SEM)

Nitrogen fertilization significantly influenced the structural properties of biopolymeric films formulated from cactus mucilage. Films produced with 50 and 450 kg N ha^–1^ exhibited favorable characteristics for applications in the biopolymeric materials industry, such as homogeneity, characterized by smooth, flexible, and easily handled surfaces (Figure 5A,J). Furthermore, at the microscopic level, these films displayed surfaces with fewer pores and cracks, indicating better compatibility among the material’s components. In contrast, films derived from mucilage fertilized with intermediate doses (150 and 300 kg N ha^–1^) were more rigid, exhibiting irregular morphologies with visible heterogeneity and brittleness (Figure 5D and G), despite being thicker (>0.44 mm) (Table 2), and thus demonstrating inadequate structural properties. Gheribi et al.^25^ and Mannai et al.^49^ reported thinner films from *O. ficus-indica* mucilage cultivated without nitrogen fertilization, measuring 0.18 mm and 0.15 mm, respectively.

**Figure 5:**
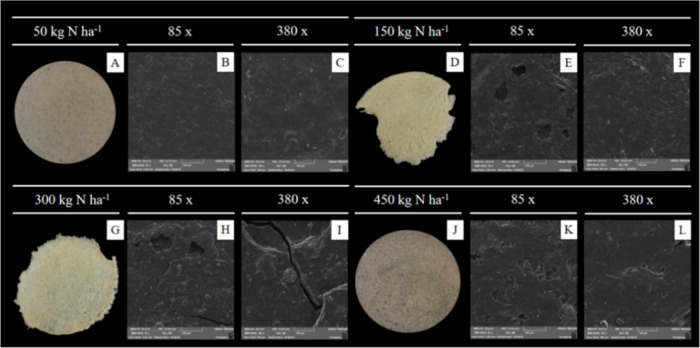
Macrographs (A, G, D, J) and micrographs at 85× (B, H, E, K) and 500× (C, I, F, L) magnifications of the surface of films based on the mucilage of *Opuntia stricta* (Haw.), subjected to nitrogen fertilization at 50 (A–C), 150 (D–F), 300 (G–I) and 450 (J–L) kg N ha^–1^.

The differences in film quality could be attributed to variations in mucilage chemical composition resulting from different nitrogen doses. The mechanism by which nitrogen availability influences polysaccharide branching and cross-linking is closely linked to the plant’s carbon–nitrogen metabolic balance. Nitrogen regulates the synthesis of amino sugars and structural proteins, as well as the activity of glycosyltransferases responsible for assembling heteropolysaccharides in the cell wall.^50^ When nitrogen levels are high, carbon skeletons are preferentially directed toward amino acid and protein synthesis rather than polysaccharide formation, which may reduce chain elongation or decrease the degree of branching.^51^ Conversely, under low nitrogen supply, plants often allocate a greater proportion of fixed carbon to polysaccharide biosynthesis, promoting the formation of more structured and better-organized polymers.^52^ These shifts can alter intermolecular interactions, cross-link density, and the ability of mucilage to form cohesive film networks.

Considering that fertilization increased carbohydrate content, these polysaccharides may be responsible for the greater thickness observed in the films. Polysaccharides present in the mucilage are the main structural components of biopolymeric films, contributing to the formation of a three-dimensional matrix that can be denser and more uniform with higher carbohydrate content, which may have increased the film thickness.^53^

#### 3.2.5 Mechanical Properties

The mechanical properties of food packaging are crucial because they affect the material’s physical integrity, directly influencing food preservation during storage and distribution.^54^ Films produced with nitrogen fertilization rates of 50 and 450 kg N ha^–1^ exhibited higher Young’s modulus values (>8.8 MPa) (Figure 6C), indicating a greater capacity to resist elastic deformation under applied external forces. Tensile strength (>0.7 MPa) (Figure 6A) and elongation at break (>11%) (Figure 6B) tended to increase with nitrogen fertilization, suggesting that fertilization promotes the formation of more flexible materials that are less prone to breakage under stress.

**Figure 6:**
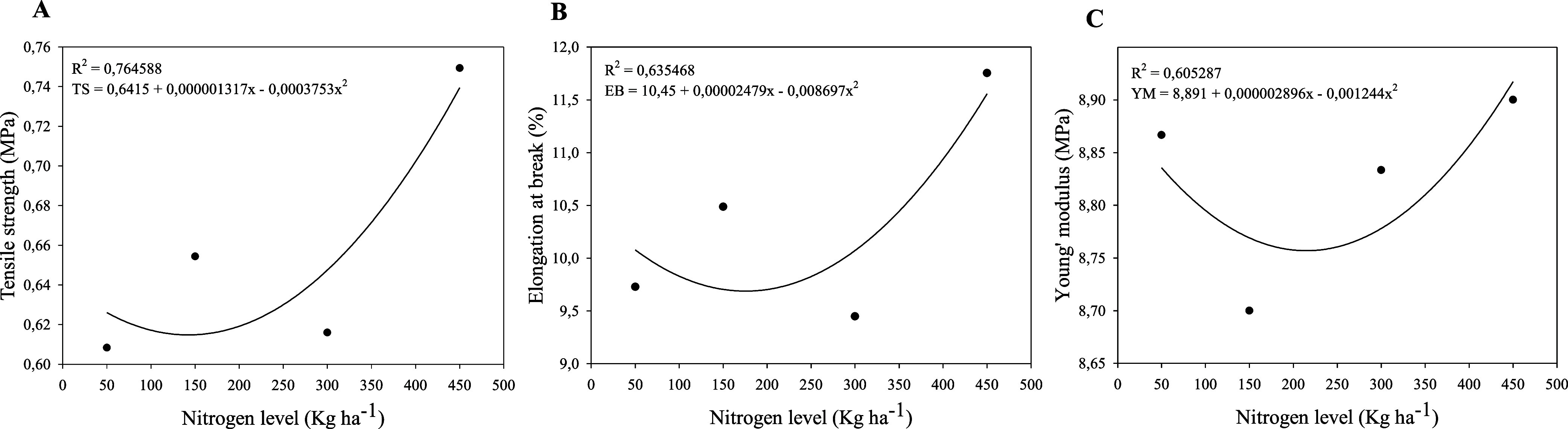
Tensile strength (MPa) (A), elongation at break (%) (B) and Young’s modulus (MPa) (C) of films based on the mucilage of *Opuntia stricta* (Haw.), subjected to nitrogen fertilization at 50, 150, 300 e 450 kg N ha^–1^.

The comparatively low tensile strength values obtained for the *O. stricta* films are consistent with the intrinsic mechanical behavior reported for films produced exclusively from cactus mucilages. This weakness is attributed to the highly branched, amorphous, and strongly hydrophilic nature of cactus heteropolysaccharides, which limits chain alignment, crystallinity, and intermolecular cohesion,^42^ features typically associated with higher tensile performance in starch-, chitosan-, or PVA-based films. Similar low mechanical values have been documented in films from *Opuntia*, such as those reported by Pinheiro et al.,^5^ who found tensile strengths typically ranging from 0.5 to 1.2 MPa in mucilage-based films.

The tensile strength and elongation values were also comparable to those reported by Gheribi et al.^25^ and Espino-Díaz et al.^27^ for *O. ficus-indica* films formulated with the same plasticizer used here, glycerol. However, these mechanical parameters were significantly lower than those of mucilage–poly­(vinyl alcohol) (PVA) blend films, which exhibited tensile strength and elongation at break values of 2.7 MPa and 55%, respectively.^42^ This suggests that incorporating additives, such as cross-linkers, nanoparticles, and hydrophobic compounds, along with forming polymer blends by mixing thermodynamically compatible polymers, can significantly enhance the mechanical properties of mucilage-based films. This approach enables the development of composite materials with desirable characteristics unattainable using individual polymers alone. Therefore, future studies are recommended to investigate the effect of nitrogen addition on polymer blends of *Opuntia* mucilage formulated with other additives and polymers.

#### 3.2.6 Water Angle Contact

The results indicated that mucilage films exhibited similar contact angle values regardless of the different nitrogen fertilization doses applied to the forage cactus cladodes (Figure 7). The minimal variation in contact angle suggests that nitrogen fertilization did not significantly affect the surface hydrophobicity or hydrophilicity of the formulated films.

**Figure 7:**
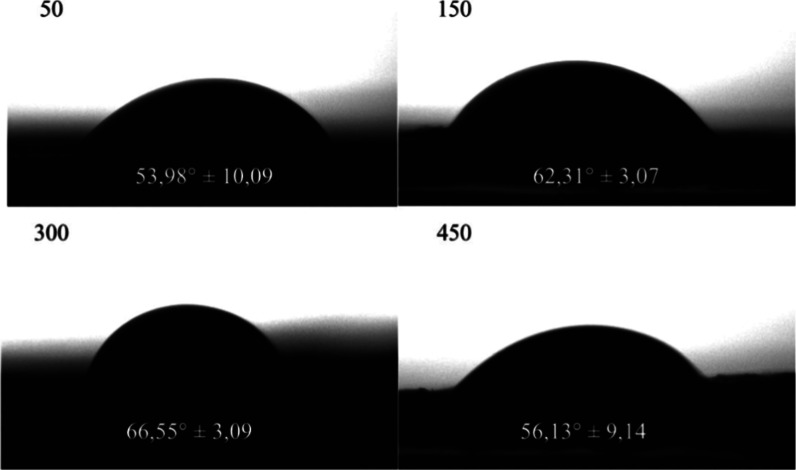
Water angle contact of films based on the mucilage of *Opuntia stricta* (Haw.), subjected to nitrogen fertilization at 50, 150, 300 e 450 kg N ha^–1^.

Contact angle measurements with water reflect the surface affinity of the films, indicating their hydrophobicity. Higher contact angle values correspond to greater water repellency and improved moisture barrier properties.^42^ The produced films exhibited relatively low contact angles (<67°) (Figure 7), indicating more hydrophilic surfaces regardless of the nitrogen dose applied. This result may be attributed to the high content of hydrophilic compounds in the mucilage, particularly polysaccharides. Additionally, rough surfaces can lead to higher contact angles compared to smooth surfaces,^50^ which may explain the higher angles observed in films from 150 and 300 kg N ha^–1^, as these films displayed rough surfaces confirmed by SEM images (Figure 5). These findings suggest that the films are more prone to water absorption, which is disadvantageous for applications requiring moisture resistance, such as food packaging. However, films from 300 kg N ha^–1^ exhibited a higher contact angle, indicating greater hydrophobicity and enhanced water-repelling capacity.

Considering the promising results obtained with the lowest nitrogen fertilization dose, it is important to explore sustainable alternatives that can replace or complement chemical fertilizers. The goal is to reduce reliance on chemical inputs while improving the economic and environmental viability of biopolymer production. A promising direction for future research is the use of rhizobia or mycorrhizal fungi in forage cactus cultivation. These microorganisms can sustainably promote plant growth by enhancing nutrient absorption and reducing the need for nitrogen fertilizers. Furthermore, the incorporation of surface-modifying agents or hydrophobic additives, such as waxes or saturated fatty acids, can help reduce the hydrophilicity of biopolymeric films, thereby expanding their industrial applications.

Exploring alternative processing techniques and optimizing formulation conditions can significantly enhance the mechanical and functional properties of biopolymeric films derived from cladode mucilage. These approaches may include adjusting drying parameters, varying plasticizer concentrations, and incorporating nanoparticles to improve mechanical strength and water vapor barrier performance. By implementing these strategies, biopolymeric films produced from forage cactus mucilage are expected to achieve superior performance, thereby increasing their potential for applications in food packaging and related fields.

## 4 Conclusions

Cladodes subjected to the lowest nitrogen doses exhibited mucilage synthesis with characteristics favorable for the formulation of biopolymeric films, including higher yield, lower pH and electrical conductivity, and reduced sodium, potassium, and water retention capacity. Moreover, films derived from plants fertilized with low nitrogen doses demonstrated superior visual and mechanical properties, such as greater transparency, flexibility, and malleability, making them promising candidates for use as biodegradable coatings. Therefore, considering the costs associated with nitrogen fertilization, applying reduced doses may offer an economic advantage in the production of mucilage-based films and coatings.

Since the highest yields were obtained from mucilage extracted from cacti fertilized with the lowest nitrogen dose, and films derived from this mucilage exhibited more compact structures, it can be concluded that efficient biopolymer production is achievable with reduced fertilizer use. This approach not only results in significant cost savings on fertilizers but also minimizes the environmental impact associated with excessive chemical fertilizer application. For future research, it is recommended to investigate the use of rhizobia or mycorrhizal fungi in cactus cultivation as alternatives to nitrogen fertilizers. These microorganisms could potentially increase nitrogen availability in the soil naturally, promoting plant growth and enhancing mucilage properties while contributing to more sustainable agricultural practices.
